# From Internet to Artificial Intelligence (Al) Bots: Symbiotic Evolutions of Digital Technologies and e-Patients

**DOI:** 10.2196/68911

**Published:** 2025-06-04

**Authors:** Daniel Z Sands, Nancy B Finn

**Affiliations:** 1 Society for Participatory Medicine, Newton, MA, United States,; 2 Beth Israel Deaconess Medical Center, 330 Brookline Ave., Boston, MA, 02215., United States, 1 6172564775; 3 Harvard Medical School, Boston, MA, United States

**Keywords:** e-patient, participatory medicine, digital health technologies, artificial intelligence in health care, patient-generated health data, electronic health records, patient portals, telemedicine or telehealth, social networking in health, smartphones and health apps, internet and health care, health care innovation, digital communication tools, self-monitoring devices, health care cost transparency, chronic disease management, OpenNotes, 21st Century Cures Act, social media in health care, consumer health informatics, data sharing, wearable electronic devices

## Abstract

This paper will view the rise of the e-patient, who is “equipped, enabled, empowered, and engaged” through the lens of the evolution of successive digital technology innovations, each building on its predecessors, creating new tools for patient empowerment. We begin with the dawn of the web and the proliferation of health websites and discuss the use of digital communication tools. We then discuss the adoption of electronic health records, which enabled the rise of patient portals. This digitization of health data, along with the rapid adoption of mobile internet access and the proliferation of health-related smartphone apps, in turn, provided a platform for patients to coproduce health care by contributing their own health data to their self-care and health care. The exchange of health information between patients and providers has also been facilitated by telehealth or telemedicine technology, which enables direct care delivery. The use of social networks in health, in use since the early days of the web, has expanded since COVID-19, when public health authorities worldwide, as well as patients, sought the use of social media channels to get connected and share information. Most recently, artificial intelligence and large language models have emerged with yet untapped potential to provide patients with the information that could improve their understanding of their conditions and treatment options. We conclude that innovations in digital health technology have symbiotically evolved with the ascendance of the e-patient, enabling improved communication, collaboration, and coordination between patients and clinicians and forging a health care system that is safer and more responsive to patient needs.

## Introduction: The Rise of the e-Patient

Until the later half of the 20th century, the concept of an empowered, engaged patient did not exist. Physicians were viewed as experts who, based on their medical education, were supposed to understand every issue or concern a patient presented. The patient was expected to comply and follow their doctor’s orders passively. Dr Tom Ferguson, physician, author, educator, and innovator, had a different view, possibly inspired by his involvement in the patient self-care movement that started in the 1970s.

In his sentinel white paper, “e-Patients: How they can help us heal health care,” completed posthumously by the e-Patient Scholars Working Group in 2007, the term e-patient is defined [1]:

*e-Patients represent the new breed of informed health consumers who go online to seek information on their own ailments and to find better health information and services for others. They work collaboratively with their doctors and within the system to resolve health issues*.

The e-Patient Scholars Working Group fostered the movement of participatory medicine, in which patients, using digital health tools, become active drivers of their health, leveraging newly developed and available digital health technologies that have changed medicine forever.

The rise of digital health technologies has fueled the emergence of the e-patient. First, the World Wide Web, followed by the adoption of electronic health records (EHRs), patient portals, and connected self-monitoring instruments that enable patient-generated health data (PGHD) and facilitate patient involvement in their own care have successively empowered patients. In addition, technologies such as smartphones, telehealth, and social networking, and finally, recent innovations that include various iterations of artificial intelligence (AI), have fostered engagement of both patients and clinicians in a way that has changed how health care operates. Pressure from patients who want to manage their own health, participate in their health decisions, communicate and collaborate with their health care providers, and push back against a health care system that does not meet their needs has led to the creation of digital technologies—with their attendant questions about safety and privacy—that have evolved to meet these needs. The rise of the e-patient and these digital technologies has shaped a new dynamic in health that has indelibly changed the face of health care and “enhanc[ed] the capacity of [patients] to make purposive choices and to transform those choices into desired actions and outcomes” [2]. We will look at 9 important innovations in recent decades and identify specifically how they have empowered patients to better pursue their health goals (Table 1).

**Table 1.** Technologies and their impact on e-patients.

Technology	e-Patient impact
World Wide Web	Web-based health information Medical literature search
Email	Patient-patient communication Patient-clinician communication
Social networking	Emotional support Sharing disease-specific information Sharing treatment and outcome data
Electronic health records	Enhanced safety Increased confidence in care
Patient portals	Direct access to medical records Communication with the clinical team Convenience transactions (appointments, prescriptions, referrals, and financial) Health information
Smartphones	Ubiquitous access to health information, portals, and social networks Health apps Health monitoring
Patient-generated health data	Insights into lifestyle and impact on health conditions Greater participation in care
Telemedicine	Improved access to professional care Access to lifestyle medicine providers “Digital primary care”
Artificial intelligence	Greater understanding of medical records Enhance comprehension of medical literature Assist with triage and diagnosis Discuss treatment options Aid to communication Gain new insights from self-monitoring data combined with medical record

## The Internet and the World Wide Web

### Overview

The internet is a global network of servers and networks originally conceived and developed to meet the demand for automated information-sharing between scientists in universities and institutes throughout the world [3]. The protocols that enabled the evolution of the World Wide Web were created by Berners-Lee et al [4]. By the mid-1990s, the proliferation of websites and the technologies for publishing on the web had democratized access to information and communication on the internet. Over the last 3 decades, there has been significant innovation in the use of the web as a platform for accessing enormous multimedia information resources and enabling many of the technologies described in this paper. The widespread adoption of these technologies has been facilitated by the development of broadband internet access, Wi-Fi, wireless internet access, and powerful and highly portable mobile technologies.

A recent Pew Research Center survey of 5733 US adults, published in January 2024, reported that nearly 95% of US adults are using the internet; 80% say they subscribe to high-speed internet (broadband) at home. The study determined that a large proportion of American people are connected to the world of digital information while “on the go” via their smartphones and other mobile devices. From these numbers, it is apparent that the internet is a staple of the 21st-century lifestyle and an important way that patients remain empowered and armed with the information and tools they need to make medical decisions [5].

### Impact of the Web on Patient Empowerment

The advent of the web has greatly facilitated patient access to health information, once largely the domain of health care professionals. A proliferation of sites provided medical information to patients, with still-running WebMD [6], which debuted in 1996, one of the earliest examples. As website technology matured, these sites offered increasing interactivity to patients to better address their questions and concerns. Interestingly, patient use of web-based information has often been opposed by the medical establishment [7], leading to conflict in patient-physician interactions. Another important example is enabling patients to search medical journals. The world’s medical literature is cataloged by the National Library of Medicine (NLM) and, beginning in 1879, a comprehensive bibliography was published on paper as Index Medicus [8]. Medical librarians and appropriately trained physicians could query this index on the NLM’s computers through MEDLINE [9] beginning in 1971. In 1986, the Grateful Med app eased access for health care professionals [10], but the advent of the web enabled the NLM to create PubMed [11], which made it easy for anyone (including patients and nonprofessional caregivers) to search the world’s biomedical literature to help diagnose and manage their medical conditions.

## Email

### Overview

Email, asynchronous computer-based communication technology, was created in the 1970s, and its use proliferated with the dawn of the web in the 1990s. In 1998, Kane and Sands [12] first promoted the broad use of email between patients and physicians and offered guidelines for its appropriate use. Prior to the use of email, only synchronous communication in the office or over the phone was used in health care interactions.

Common uses of patient-provider email are many and include advice regarding new or recurrent medical conditions, including recommendations on the best site of care (home vs clinic vs urgent care vs emergency department), which may include photos or other media as needed; response to quick questions that should not involve an office visit; sharing data such as blood pressure and blood sugar; and follow-up on the effectiveness or side effects of medications.

Because of the need for patient privacy, which is not inherent in email, patient portals, offering secure messaging, gained widespread use in the 2010s. Many of these messages today are triaged by nursing staff before being sent to physicians.

### Impact of Email on Patient Empowerment

AIDS activists used email for information sharing and organizing in the 1980s. Patient-physician email broke down communication barriers imposed by phone-based triage and “telephone tag” and permitted a greater frequency of brief connections, thereby potentially enhancing relationships. Because it is asynchronous, it removes the time pressure of the office visit, affording patients the ability to take the time to craft their questions and more time to absorb their physicians’ responses [13].

## Social Networking

### Overview

Although many think of social networking as a recent phenomenon, early social networks, such as USENET, FIDONET, and The WELL, date to the 1980s and enabled mainly asynchronous communication on a variety of topics. The advent of the web and faster connection speeds enabled the immersive social networking experience to which we have become accustomed. These platforms permit peer-to-peer information-sharing and support.

### Impact of Social Networks on Patient Empowerment

e-Patients do not rely on medical professionals’ views alone. Not surprisingly, in the 1980s, they began actively engaging with peers to share information and support through health groups on USENET, FIDONET, and The WELL. These became popular for AIDS activists to share information and support [14,15]. Peer-support communities proliferated in the early days of the web. For example, in 1995, the Association of Cancer Online Resources began to offer cancer-specific support for patients with cancer and their caregivers, ultimately offering communities for more than 200 different cancers with 115,000 messages exchanged each day [16]. Frydman (personal communication, 2025), the founder of the Association of Cancer Online Resources, estimates that the site helped over half a million people. Over the subsequent years, web-based health communities proliferated and were a primary source of information during the COVID-19 pandemic. Many web-based peer-support networks bring together patients who are living with illnesses and health care professionals who may be interested in these conditions.

There are web-based communities for different cancers, neurologic diseases, autoimmune diseases, mental health disorders, and many other conditions. These communities provide emotional support, peer coaching, and medical advice. The advice gathered from these communities has been reported to be life-saving [17]. Like other forms of web-based information, individuals in communities may provide incorrect advice. Studies show that communities will usually self-correct erroneous information [18].

While these and their successors were generally platforms for peers to share emotional and care advice, in 2004, PatientsLikeMe created a web-based community health data platform that also encouraged patient-driven research collaboration to test therapies and share actual outcome data [19]. The network has over 800,000 members who are dealing with more than 2900 conditions, including amyotrophic lateral sclerosis, multiple sclerosis, and epilepsy [20]. As the technology has improved, web-based support communities have added synchronous tools like chat and video, and in some cases, have facilitated patient meet-ups in real life [21].

## Electronic Health Records

### Overview

Digital health records got off to a slow start when they were introduced in the United States starting in the 1980s. It was not until 2004, when President George Bush set the goal that every American would have an EHR within 10 years, supported with funding for demonstration projects and the development of common standards that digital health records became ubiquitous [22]. The passage of the Health Information Technology for Economic and Clinical Health Act, enacted under Title XIII of the American Recovery and Reinvestment Act of 2009, helped to foster the growth of the EHR. In 2008, only 17% of health care providers had electronic medical records, but by 2021, 9 in 10 US office-based physicians had adopted EHRs [23].

### Impact of EHRs on Patient Empowerment

Even before the advent of patient portals, the adoption of EHRs may have led to greater patient confidence in the safety of their care and the persistence of their health data and reduced frustration when they see the availability of their health records to all their physicians. However, the greater impact was yet to come when patient-facing apps were added to their physicians’ EHRs in the form of patient portals.

## Patient Portals

### Overview

EHRs were adopted to improve the quality and safety of patient care, but they also permitted patients access to their health information through connected patient portals. Patient portals are secure websites that provide access to EHR information (including sharing access with caregivers), communication with the health care team, and convenience transactions such as tools for booking appointments, requesting prescriptions, and paying medical bills. Through these portals, patients can view substantial parts of their medical records—including office notes, thanks to the advocacy of organizations like OpenNotes [24]—pulling back the curtain on health care decision-making and permitting them to manage and monitor their health issues and collaborate with their physicians to resolve health problems.

### Impact of Portals on Patient Empowerment

Patient portals have had a major impact on patients’ ability to engage in their health care. For one, portals have facilitated secure asynchronous communication between patients and health care professionals, reducing barriers to communication and sometimes obviating the need for a medical appointment. It has also been a useful mechanism for patients to provide updates on their conditions, such as sharing blood pressure measurements or responses to medications. Messaging has become so popular among patients, especially since the COVID-19 pandemic, that it has been cited as a contributor to physician burnout [25].

While streamlining transactions, such as requesting prescription renewals and making appointments, has further made it easier for patients to interact with their physicians’ offices, arguably the most important impact of patient portals has been to enable patients to see their own health information. Initially, this was only problems, medications, and test results, but patients wanted more, and activists and advocacy organizations (including the Society for Participatory Medicine) pushed the Obama administration to require that patients have full access to their records.

The 21st Century Cures Act (Cures Act) [26], signed into law on December 13, 2016, was designed to help accelerate medical product development and bring innovations and advances to patients who need them faster and more efficiently. The Cures Act legislation makes patient access easier and digitally unrestricted by mandating that providers give them access to data from their medical records so they can make better choices regarding their care and experience transparency regarding costs and health care outcomes.

However, just viewing information is not enough. e-Patients want to download their data and use it in novel ways. Dedicated technology and patient activists worked together to develop the capabilities of Fast Healthcare Interoperability Resources, a data exchange standard, to support this functionality, and the Cures Act requires providers to offer an application programming interface to EHRs to permit patients to download their records, usually through apps [27]. Each of these improvements enhanced the patient’s ability to know what is going on with their health, which is the cornerstone of empowerment.

## The Smartphone

### Overview

Modern smartphones combine a full suite of mobile tools for patients and clinicians in one compact device that has a large memory, fast processing speeds, wireless internet access (both through the mobile networks and Wi-Fi), a high-quality camera, an accelerometer, GPS, Bluetooth for connectivity to devices, near-field communication, and, of course, a phone. They provide the ability to manage personal information, streaming music, videos, and games, 24/7 access to social media, text messaging, and real-time language translation. The number of tasks that can be accomplished with this platform is almost infinitely expandable through access to app stores. The average person uses 9 mobile apps daily, 30 apps per month [28].

A Pew Research study in 2023 [5] found that 90% of adults reported they owned a smartphone, and 4 in 10 individuals polled reported being on the web “almost” constantly. The study found that smartphones are used across income levels, but those in households earning US $100,000 or more annually are far more likely than those earning less than US $30,000 per year to use a smartphone (98% vs 79%). Education level and age also played a factor in the ownership of smartphones. Those individuals with a higher education generally had a smartphone. People older than 65 years of age were reported to be about 20% less likely to have a smartphone than those younger than 50 years.

### Impact of Smartphones on Patient Empowerment

Smartphones provide patients with ubiquitous access to health information, including their health records, participation in social networks, connection with their health care team, health plan, and pharmacy, as well as access to apps that allow them to track their activity, food intake, blood pressure, glucose, sleep, and weight. Combined with connected wearable devices like smartwatches, available apps can also track heart rate and rhythm, oxygen saturation, and cardiovascular fitness. Being better informed about their health status and better equipped to take timely action empower patients to better manage their health between visits. App stores host more than 350,000 health care–related apps available globally, and new health apps are constantly being developed.

## Patient-Generated Health Data

### Overview

According to the RAND Corporation, nearly 60% of adult American people have at least 1 chronic disease—including diabetes, cardiovascular diseases, such as irregular heart rhythm or hypertension, or lung problems such as asthma or chronic obstructive pulmonary disease, cancer, arthritis, and kidney disease—and 42% have more than 1 [29]. These chronic conditions account for hundreds of billions of dollars in health care spending every year in the United States alone. Their estimates suggest that nearly 150 million American people are living with at least 1 chronic condition; around 100 million of them have more than 1. Nearly 30 million are living, day in and day out, with 5 chronic conditions or more.

In a 2019 study of 4159 individuals from the Health Information National Trend Survey [30], about 30% were using a wearable device. The use of wearable devices was more common among those with chronic conditions. This study found that 49% of those with a usual source of care had shared data with their provider. This behavior was more common in those with chronic conditions. Both adoption and data sharing have likely risen in the ensuing years.

Since patients only spend a small fraction of their lives in formal medical care, PGHD have increasing potential to help patients with self-care and improve the health care of patients with many chronic conditions. In their 2014 paper on the topic, Sands and Wald concluded [31]:

*Patient-generated health information, enabled by data transparency and consumer engagement, is not a panacea, but can help address information gaps in important areas, leverage untapped patient experience, and offer information that will improve self-management, provider-directed, and joint decisions made by patients and providers together and facilitate more frequent contacts with patients for better management of chronic conditions.*

### Impact of PGHD on Patient Empowerment

Home blood pressure cuffs have been in use since the 1970s, and glucometers have been used widely since the 1990s. Both technologies have enabled patients to contribute data to their care and self-care, improving their self-awareness and enriching the data available to their clinicians.

Although electronic biometric self-tracking dates back to the 1970s, the availability of a new generation of wearable devices caught the attention of Kelly and Wolf [32] at *Wired Magazine*, who proposed the “quantified self” movement as a means to self-knowledge in 2007 [32]. Internet-connected wearable devices such as the Fitbit (2008) prompted increasing consumer demand [33], which led to ongoing innovation, and ultimately the incorporation of multifunction self-tracking into wearable devices in the form of a watch [34] and even a ring [35]. e-Patients have been able to leverage successive generations of self-tracking technologies for their self-care and to share this information with their physicians, while companies have developed apps to facilitate structured data sharing.

In another vein, patients with type 1 diabetes, dissatisfied with the state of siloed diabetes technology and unified by the hashtag #WeAreNotWaiting, developed a do-it-yourself closed-loop system in 2014 that integrates data from continuous glucose monitors with their insulin pumps to better manage their diabetes [36]. Commercial entities later developed their own systems based on that e-patient innovation.

## Telemedicine or Telehealth

### Overview

The convergence of the internet, high-speed telecommunications, video technology, and the availability of patients’ digital health records make it possible for real-time video visits between a clinician and a patient to occur over a remote network on a computer screen or smartphone. Telemedicine consultations can be augmented with PGHD to address the difficulty of telemedicine physical examinations. With PGHD and a patient history, the examining physician will have baseline information. This is a viable option for patients in need of medical assistance, and although the physical examination is quite limited, there are guidelines that physicians can use to do physical examinations via telemedicine [37].

For many years, telemedicine struggled with slow adoption, partly due to a lack of payment for services rendered remotely and partly due to the lack of infrastructure to conduct such video calls. The COVID-19 pandemic prompted payers to change their payment policies to encourage telemedicine encounters; telemedicine use increased from 11% to over 60% in a very short time [38]. After the pandemic, reimbursement for telehealth remains in place, as it has been remarkably popular. As health care has become more digitized, physicians across specialties are integrating telemedicine into their practices. A remaining obstacle is that almost all state medical boards continue to prohibit care of patients within that state by physicians not licensed in that state [39].

### Impact of Telemedicine on Patient Empowerment

Patients have been the beneficiaries of the wider use of telemedicine, and patient demand for remote care has mirrored workers’ demand for remote work. This has resulted in greater technological innovation, as it has spawned a rising number of businesses, and business models focused on meeting the rising demand for remote care. For example, the need for mental health care has far exceeded the availability of local therapists, so numerous companies are providing “telemental health” services. Numerous companies are providing direct-to-consumer remote care for “lifestyle” health needs, such as sexual health, hair growth, and weight management. Finally, the shortage of primary care physicians has prompted the development of “digital primary care,” which was pioneered in Sweden [40] and is being promoted in the United States as an alternative to traditional primary care.

## Artificial Intelligence

### Overview

A few years ago, physicians made medical decisions based on the knowledge they accumulated during their training and subsequent experience. Today, the rapid development of AI is slowly changing that. Machine learning can process vast amounts of information to identify hidden patterns and replicate clinical thought processes. AI and machine learning are increasingly used in fields such as pathology, radiology, and gastroenterology [41,42]. The advent of chatbots, such as ChatGPT, Gemini, and Claude, built on large language models, has profoundly changed how we search for and interact with information, including health information.

More importantly, for patients, though, the availability to consumers (patients) of generative AI has produced an explosion in patient access to advanced clinical information. In the words of Dave deBronkart, as quoted in the *New York Times* [43]: “Google gives you access to information. A.I. gives access to clinical thought.”

### Impact of AI on Patient Empowerment

AI chatbots have been a boon for patients (as well as health care professionals), allowing them to better understand their health conditions, not only by answering questions but also by helping them understand their medical records [44-46]. These tools have enabled patients to diagnose conditions when their physicians have been unable to do so, underscoring the empowering nature of having access to clinical reasoning [47]. Leveraging AI, patients can combine large quantities of self-tracking data and data from their medical records to gain new insights into their health [48], leading to proposals for responsible governance [49]. The future uses of these technologies will continue to expand, pushed by technology-savvy e-patients.

## Conclusions

We have witnessed exponential advancements in communication and information technology followed by their rapid adoption. e-Patients use these technologies to learn about, get support for, obtain care for, and manage their health and illnesses. e-Patients, many of whom are impatient and frustrated with the status quo, will spur technological innovation, sometimes even developing technologies themselves.

We are at the precipice of dramatic transformations in health care made possible by the expanding capabilities and availability of AI, machine learning, communication, and self-monitoring technologies. This revolution is timely, as we confront an aging population, a proliferation of chronic diseases, and a shortage of health care professionals.

We must be considerate about introducing any technology, but AI presents unique ethical challenges. Concerns regarding patient safety, quality, and data privacy and security, along with the stability of different care models that prioritize equity and inclusion at an affordable cost, are all crucial questions that currently lack satisfactory answers. We anticipate that as digital health technologies continue to evolve, e-patients will continue to leverage these technologies to facilitate self-care and improvements in their health care experiences, which will, in turn, spur the evolution of the next generation of digital health technologies.

## Abbreviations

AI: artificial intelligence
EHR: electronic health record
NLM: National Library of Medicine
PGHD: patient-generated health data

## References

[1] Ferguson T, e-Patient Scholars Working Group (2007). e-patients: how they can help us heal health care. Society for Participatory Medicine.

[2] (2007). Empowerment in practice: analysis and implementation. https://openknowledge.worldbank.org/server/api/core/bitstreams/43f6c98e-646c-5caa-bf46-2cae86e82a7c/content.

[3] Leiner BM, Cerf VG, Clark DD (2009). A brief history of the internet. SIGCOMM Comput Commun Rev.

[4] Berners-Lee T, Cailliau R, Luotonen A, Nielsen HF, Secret A (1994). The World-Wide Web. Commun ACM.

[5] Gelles-Watnick R (2024). Americans’ use of mobile technology and home broadband. Pew Research Center.

[6] Better information better health. WebMD.

[7] Ahmad F, Hudak PL, Bercovitz K, Hollenberg E, Levinson W (2006). Are physicians ready for patients with internet-based health information?. J Med Internet Res.

[8] Kunz J (1979). Index Medicus. A century of medical citation. JAMA.

[9] Arnott Smith C (2005). An evolution of experts: MEDLINE in the library school. J Med Libr Assoc.

[10] Lindberg DA (1996). The NLM and Grateful Med: promise, public health, and policy. Public Health Rep.

[11] (1997). Free web-based access to NLM databases. NLM Technical Bulletin.

[12] Kane B, Sands DZ (1998). Guidelines for the clinical use of electronic mail with patients. The AMIA Internet Working Group, Task Force on Guidelines for the Use of Clinic-Patient Electronic Mail. J Am Med Inform Assoc.

[13] Delbanco T, Sands DZ (2004). Electrons in flight—e-mail between doctors and patients. N Engl J Med.

[14] McKinney C (2018). Printing the network: AIDS activism and online access in the 1980s. Continuum (Mount Lawley).

[15] Makulowich JS (1992). AIDS and electronic communications. AIDS Patient Care.

[16] Eysenbach G (2003). The impact of the internet on cancer outcomes. CA Cancer J Clin.

[17] deBronkart D (2013). How the e-patient community helped save my life: an essay by Dave deBronkart. BMJ.

[18] Esquivel A, Meric-Bernstam F, Bernstam EV (2006). Accuracy and self correction of information received from an internet breast cancer list: content analysis. BMJ.

[19] Wicks P, Massagli M, Frost J (2010). Sharing health data for better outcomes on PatientsLikeMe. J Med Internet Res.

[20] (2025). About us. PatientsLikeMe.

[21] Gavrila V, Garrity A, Hirschfeld E, Edwards B, Lee JM (2019). Peer support through a diabetes social media community. J Diabetes Sci Technol.

[22] Bush GW (2004). A new generation of american innovation. https://georgewbush-whitehouse.archives.gov/infocus/technology/economic_policy200404/chap3.html.

[23] Powers G (2013). The power of incentives: the story of electronic medical records. Center for Health & Research Transformation.

[24] Walker J, Leveille S, Bell S (2019). OpenNotes after 7 years: patient experiences with ongoing access to their clinicians’ outpatient visit notes. J Med Internet Res.

[25] Stillman M (2023). Death by patient portal. JAMA.

[26] (2016). 21st Century Cures Act: 114th Congress. Congress.Gov.

[27] Gordon WJ, Mandl KD (2020). The 21st Century Cures Act: a competitive apps market and the risk of innovation blocking. J Med Internet Res.

[28] Number of apps available in leading app stores as of August 2024. Statista.

[29] Buttorff C, Ruder T, Bauman M (2017). Multiple chronic conditions in the United States. RAND Corporation.

[30] Turner K, Jo A, Wei G, Tabriz AA, Clary A, Jim HSL (2021). Sharing patient-generated data with healthcare providers: findings from a 2019 national survey. J Am Med Inform Assoc.

[31] Sands DZ, Wald JS (2014). Transforming health care delivery through consumer engagement, health data transparency, and patient-generated health information. Yearb Med Inform.

[32] Kelly K, Wolf G (2011). What is the quantified self?. The Quantified Self.

[33] Swan M (2013). The quantified self: fundamental disruption in big data science and biological discovery. Big Data.

[34] Rawassizadeh R, Price BA, Petre M (2015). Wearables: has the age of smartwatches finally arrived. Commun ACM.

[35] Fiore M, Bianconi A, Sicari G (2024). The use of smart rings in health monitoring—a meta-analysis. Appl Sci (Basel).

[36] Palmer W, Greeley SAW, Naylor R (2021). The do-it-yourself artificial pancreas. Pediatr Ann.

[37] Benziger CP, Huffman MD, Sweis RN, Stone NJ (2021). The telehealth ten: a guide for a patient-assisted virtual physical examination. Am J Med.

[38] Bakalar RS, Kiel JM, Kim GR, Ball MJ (2022). Healthcare Information Management Systems: Cases, Strategies, and Solutions.

[39] Raghu G, Mehrotra A (2023). Licensure laws and other barriers to telemedicine and telehealth: an urgent need for reform. Lancet Respir Med.

[40] Ekman B (2018). Cost analysis of a digital health care model in Sweden. Pharmacoecon Open.

[41] Hunter B, Hindocha S, Lee RW (2022). The role of artificial intelligence in early cancer diagnosis. Cancers (Basel).

[42] Nassif AB, Talib MA, Nasir Q, Afadar Y, Elgendy O (2022). Breast cancer detection using artificial intelligence techniques: a systematic literature review. Artif Intell Med.

[43] Rosenbluth T (2024). Dr. Chatbot will see you now. The New York Times.

[44] Blease C, Torous J, McMillan B, Hägglund M, Mandl KD (2024). Generative language models and open notes: exploring the promise and limitations. JMIR Med Educ.

[45] Zaretsky J, Kim JM, Baskharoun S (2024). Generative artificial intelligence to transform inpatient discharge summaries to patient-friendly language and format. JAMA Netw Open.

[46] e-Patient Dave (2023). ChatGPT summarized my opennotes. It’s so much more usable!. Society for Participatory Medicine.

[47] Goldberg C (2024). Patient Portal — When Patients Take AI into Their Own Hands. NEJM AI.

[48] Advancing personal health and wellness insights with AI. Google Research.

[49] Rozenblit L, Price A, Solomonides A (2025). Towards a multi-stakeholder process for developing responsible AI governance in consumer health. Int J Med Inform.

